# Effects of Increasing Doses of Condensed Tannins Extract from *Cistus ladanifer* L. on In Vitro Ruminal Fermentation and Biohydrogenation

**DOI:** 10.3390/ani11030761

**Published:** 2021-03-10

**Authors:** Olinda Guerreiro, Susana P. Alves, Mónica Costa, Maria F. Duarte, Eliana Jerónimo, Rui J. B. Bessa

**Affiliations:** 1 Centro de Biotecnologia Agrícola e Agro-Alimentar do Alentejo (CEBAL), Instituto Politécnico de Beja (IPBeja), 7801-908 Beja, Portugal; olinda.guerreiro@cebal.pt (O.G.); fatima.duarte@cebal.pt (M.F.D.); 2 MED-Mediterranean Institute for Agriculture, Environment and Development, CEBAL, 7801-908 Beja, Portugal; 3 Centro de Investigação Interdisciplinar em Sanidade Animal (CIISA), Avenida da Universidade Técnica, 1300-477 Lisboa, Portugal; susanaalves@fmv.ulisboa.pt (S.P.A.); admonicacosta@fmv.ulisboa.pt (M.C.); rjbbessa@fmv.ulisboa.pt (R.J.B.B.); 4 Faculdade de Medicina Veterinária, Universidade de Lisboa, Avenida da Universidade Técnica, 1300-477 Lisboa, Portugal

**Keywords:** Mediterranean shrub, phenolic compound, rumen metabolism, fatty acids, biohydrogenation intermediates, dimethyl acetals

## Abstract

**Simple Summary:**

Ruminant edible products have been associated with adverse health effects, due to their high saturated fatty acids and low polyunsaturated fatty acids content, resulting from the extensive biohydrogenation conducted by rumen microbiota. *Cistus ladanifer* condensed tannins were able to change the lamb ruminal biohydrogenation, increasing the beneficial fatty acids production. The aim of this study was to test the effect of increasing doses of *C. ladanifer* condensed tannins extract (0, 25, 50, 75 and 100 g/kg dry matter) on in vitro rumen fermentation and biohydrogenation. The increasing doses of condensed tannins led to a moderate decrease of volatile fatty acids production, a pronounced depression in microbial odd and branched fatty acids and of dimethyl acetals production, and a minor effect on the biohydrogenation, which indicates that microbial growth was more inhibited than fermentative and biohydrogenation activities. The ability of *C. ladanifer* condensed tannins extract to modulate the biohydrogenation (BH) was not observed in the present study. However, the results obtained suggest a possible adaptative response of the microbial population to stress stimuli of condensed tannins and lipid supplementation.

**Abstract:**

*Cistus ladanifer* (rockrose) is a perennial shrub quite abundant in the Mediterranean region, and it is a rich source in secondary compounds such as condensed tannins (CTs). Condensed tannins from *C. ladanifer* were able to change the ruminal biohydrogenation (BH), increasing the *t*11–18:1 and *c*9,*t*11–18:2 production. However, the adequate conditions of the *C. ladanifer* CTs used to optimize the production of *t*11–18:1 and *c*9,*t*11–18:2 is not yet known. Thus, we tested the effect of increasing the doses of *C. ladanifer* CT extract (0, 25, 50, 75 and 100 g/kg dry matter (DM)) on in vitro rumen BH. Five in vitro batch incubations replicates were conducted using an oil supplemented high-concentrate substrate, incubated for 24 h with 6 mL of buffered ruminal fluid. Volatile fatty acids (VFAs) and long chain fatty acids (FA) were analyzed at 0 h and 24 h, and BH of *c*9–18:1, *c*9, *c*12–18:2 and *c*9, *c*12, *c*15–18:3, and BH products yield were computed. Increasing doses of *C. ladanifer* CTs led to a moderate linear decrease (*p* < 0.001) of the VFA production (a reduction of 27% with the highest dose compared to control). The disappearance of *c*9–18:1 and *c*9,*c*12–18:2 as well as the production of *t*11–18:1 and *c*9, *t*11:18:2 was not affected by increasing doses of *C. ladanifer* CTs, and only the disappearance of *c*9, *c*12, *c*15–18:3 suffered a mild linear decrease (a reduction of 24% with the highest dose compared to control). Nevertheless, increasing the *C. ladanifer* CT dose led to a strong depression of microbial odd and branched fatty acids and of dimethyl acetals production (less than 65% with the highest dose compared to control), which indicates that microbial growth was more inhibited than fermentative and biohydrogenation activities, in a possible adaptative response of microbial population to stress induced to CTs and polyunsaturated fatty acids. The ability of *C. ladanifer* to modulate the ruminal BH was not verified in the current in vitro experimental conditions, emphasizing the inconsistent BH response to CTs and highlighting the need to continue seeking the optimal conditions for using CTs to improve the fatty acid profile of ruminant fat.

## 1. Introduction

Tannins are a group of plant secondary compounds, defined as naturally occurring water-soluble polyphenols that have the ability to precipitate proteins [1,2]. Tannins are generally classified as hydrolysable and condensed tannins (CTs). Condensed tannin polymers vary tremendously in their constituent monomers, stereochemistry, polymer size, and intermolecular linkages, in addition to their concentration [3] and are generally considered as antinutritional factors due to their adverse effects on feed intake, nutrient utilization and toxicity [4]. However, CTs have also been recognized as useful phytochemicals for modulating rumen microbial fermentation [2], and ruminal biohydrogenation (BH) of dietary unsaturated fatty acids (FAs) [5,6,7].

Ruminant edible products have been associated with adverse health effects, due to their high saturated FA (SFA) and low polyunsaturated FA (PUFA) content, due to the extensive BH conducted by rumen microbiota [8]. Therefore, manipulation of the ruminal BH has been attempted as way if improving the nutritional value of ruminant fat, by increasing the rumen outflow of the dietary PUFA and the beneficial biohydrogenation intermediates, such as vaccenic acid (*t*11–18:1) and rumenic acid (*c*9,*t*11–18:2) [9,10]. Some in vitro studies have suggested that CTs may be efficient in inhibiting the last step of BH, decreasing the 18:0 formation and leading to *t*11-18:1 accumulation [5,6,11]. However, other studies reported a general inhibition or stimulation of BH without the inhibition of its last step [7,12,13].

*Cistus ladanifer* L. is a shrub widely spread over the Mediterranean region and often used by grazing ruminants, particularly during periods of pasture scarcity [14]. The inclusion of the *C. ladanifer* plant into oil-supplemented diets modified the lamb abomasal and meat FA composition [15,16,17]. When included in an oil-supplemented high-forage diet, *C. ladanifer* induced a large increase in *t*11–18:1 in abomasal digesta and increased the deposition of *t*11–18:1 and *c*9,*t*11–18:2 in the intramuscular fat of lambs [15]. An increase in the *trans*-18:1 isomers in digestive contents (rumen and abomasum) and the intramuscular fat of lambs was also obtained with the inclusion of the *C. ladanifer* plant in oil-supplemented medium-forage diet [17,18]. *Cistus ladanifer* presents high amounts of CTs (40–160 g/kg dry matter (DM)) [14], and its effects on ruminal BH seems to be associated with action of CTs on ruminal metabolism. Comparing the effect of several secondary compound fractions present in *C. ladanifer* (essential oil, dichloromethane extract, total phenolics, non-tannin phenols and condensed tannins fractions) on in vitro rumen BH, was possible to observe that CT extract is the secondary compound fraction of *C. ladanifer,* with highest capacity to modulate the BH pattern, inducing a larger accumulation of *t*11-18:1 [7].

Such results showed that *C. ladanifer* CTs can modulate the ruminal BH and potentially improve the nutritional value of ruminant fat. However, as observed for other CT sources, the effects of CTs from *C. ladanifer* on BH might change with the dose [11,13]. Moreover, there is increasing evidence that the effect of tannins on ruminal BH depends on the features of the basal diet [19]. The ability of *C. ladanifer* CT extract to modulate the in vitro ruminal BH was only demonstrated using an oil-supplemented high-forage substrate [7,12]. Thus, the present in vitro experiment was designed to evaluate the effect of increasing doses of CT extract from *C. ladanifer* on rumen fermentation and BH when an oil-supplemented high-concentrate substrate is used. Moreover, the present study also aimed to determine the dose of *C. ladanifer* CT doses which optimize the *c*9,*t*11-18:2 and *t*11:18:1 production.

## 2. Materials and Methods

### 2.1. Cistus Ladanifer Sampling

*Cistus ladanifer* aerial parts were harvested in January 2013, in the Baixo Alentejo region, in Monte do Vento, Mértola, southern Portugal (37°48’28.17’’ N–7°40’39.08’’ W), in a parcel of holm oak forest of *Quercus rotundifolia* L., where naturally occurring *C. ladanifer* plants are the predominant vegetation. Samples were manually harvested with scissors and kept at −20 °C for 1 week until further use. The collected aerial part of plants was composed of leaves and soft stems.

### 2.2. Preparation of Cistus Ladanifer Condensed Tannins Extract

The condensed tannin (CT) extract was obtained by the sequential extraction of *C. ladanifer* aerial parts and purified using a Sephadex LH-20 chromatographic column (GE Healthcare Bio-Science, Uppsala, Sweden), as described by Guerreiro et al. [7]. Condensed tannin extract was maintained at −20 °C until in vitro incubation. Condensed tannin extract presents 600 g of CT/kg DM, determined using *C. ladanifer* purified CT as standard and following the methodology described in Guerreiro et al. [14].

### 2.3. In Vitro Incubation with Ruminal Fluid

The experimental animal procedures were approved by the Ethical and Animal Well-Being Commission (CEBEA) of the Faculty of Veterinary Medicine, University of Lisbon, Portugal (Protocol FMV/CEBEA 007/2016). All methods and procedures were performed following the established guidelines from this institution and following compliance guidelines of European Union (Directive 86/609/EEC). Additionally, authors O. Guerreiro, M. Costa, S.P. Alves and R.J.B. Bessa hold a FELASA (Federation of European Laboratory Animal Society Associations) grade C certificate that enables them to design and carry out animal experimentation under the European Union regulations.

Ruminal fluid was collected from two rumen-fistulated Merino Branco rams (40 ± 2 kg live weight), fed daily 600 g of commercial concentrate and 600 g of oat hay, in two equal meals at 9:30 and 17:00 h. The concentrate mixture comprised maize, soybean, sunflower, wheat, wheat bran and rape (220 g crude protein; 95 g crude fiber; 65 g ash; 35 g ether extract; 4 g sodium; 7500 IU of vitamin A; 1500 IU of vitamin D_3_ and 7.5 mg of vitamin E; per kg of DM). Ruminal fluid was collected just before the morning feeding in warm flasks (approximately 39 °C) and strained through four layers of gauze. Strained ruminal fluid from both rams was pooled and it was diluted with a phosphate–bicarbonate buffer solution [20], pre-warmed at 39 °C and saturated with CO_2_, in a proportion of 1:4 (ruminal fluid:buffer solution, *v/v*), under constant CO_2_ flux. Before adding the ruminal fluid, a redox indicator (resazurin solution, 0.1% (*w/v*)) and a reducing agent (625 mg of L-cysteine-HCl, 1 N of sodium hydroxide and 625 mg of sodium sulfide nonahydrate) were added to the buffer solution. Reduction was indicated upon a change in color of the blue resazurin to colorless dihydroresorufin.

After the complete reduction of the buffer solution and mixture with strained ruminal fluid, 6 mL of buffered ruminal fluid was added to Hungate tubes containing 60 mg DM of ground feed substrate (control), or 60 mg DM of feed substrate plus each dose of *C. ladanifer* CT extract (1.5, 3.0, 4.5 and 6 mg). The final concentrations of added *C. ladanifer* CT extract were 25, 50, 75 and 100 g/kg DM, corresponding to 1.5%, 3%, 4.5% and 6% of CT, respectively. The basal substrate was composed of a mixture of commercial concentrate (564 g/kg DM), grass hay (376 g/kg DM) and sunflower oil (60 g/kg DM). The commercial concentrate was the same used to feed the rumen content donors. In this work, a high-concentrate diet was used, which is a type of diet typically used in ruminant production systems, particularly in the finishing stage. Dietary supplementation with unsaturated fatty acids has been extensively tested to improve the nutritional value of ruminant fat, and vegetable oil levels up to 6% have been successfully applied in lamb and in this context without compromising the ruminal metabolism and animal productivity [15,21,22,23].

The tubes were then filled with CO_2_, thus ensuring oxygen-free conditions and closed with a butyl rubber stopper and screw cap. The tubes allocated to the 0 h incubation time were immediately frozen at −20 °C after the addition of the buffered ruminal fluid. Incubation was performed in a water-bath (Unitronic Pro, JP Selecta, Barcelona, Spain) at 39 °C with gentle agitation for 24 h. At 0 and 24 h of incubation, the pH of ruminal fluid was determined. At the end of the incubation, tubes were immediately frozen at −20 °C. For each treatment and incubation time, 2 Hungate tubes were used, one for volatile fatty acid (VFA) determination and other tube for other FA determination. The allocation of tubes to the treatments, incubation time, order of filling with buffered ruminal fluid, and to the position in the water-bath were randomized. The incubation procedure was replicated 5 times in 5 consecutive weeks.

The incubation tubes used for FA analysis were freeze-dried (ScanVac CoolSafe, LaboGene ApS, Lynge, Denmark), and stored at −20 °C until analysis. The tubes for analysis of VFA were kept at −20 °C until analysis.

### 2.4. Fatty Acids Analysis

Volatile FAs (VFAs) were analyzed by gas chromatography with flame ionization detection (GC-FID) using a Shimadzu GC-2010 Plus chromatograph (Shimadzu, Kyoto, Japan) equipped with a fused silica capillary column (Nukol, 30 m, 0.25 mm i.d., 0.25 µm film thickness, Supelco, Sigma–Aldrich, St. Louis, MO, USA), as described by Oliveira et al. [24].

The contents of each incubation tube was transesterified to prepare FA methyl esters and dimethyl acetals (DMAs) by using a combined basic followed by acid catalysis adapted from Jenkins [25] and modified by Alves et al. [26], used as the internal standard 19:0 (1 mg/mL). Fatty acid methyl esters were separated by gas chromatography using a Shimadzu GC-2010 Plus chromatograph (Shimadzu, Kyoto, Japan) equipped with a SP-2560 capillary column (100 m, 0.25 mm i.d., 0.20 µm film thickness, Supelco, Sigma–Aldrich Inc., Bellefont, PA, USA). The peak identification was based on the comparison of retention times with FA methyl esters standards (37 component FAME mix and bacterial acid methyl esters mix from Supelco, Sigma–Aldrich Inc., Bellefont, PA, USA) and by comparison with published chromatograms [26]. Identifications were confirmed by gas chromatography mass spectrometry using a Shimadzu GC-MS QP2010-Plus instrument (Shimadzu, Kyoto, Japan).

### 2.5. Calculations

The VFA and FA (except C18 FA) and DMA productions during the incubation time were calculated directly from their concentrations at 24 h minus 0 h incubation times (presented in the Supplementary Table S1). The C18 FA differences between 24 h and 0 h were computed using the mean content of the total amount of C18 FA in each pair of tubes (presented in the Supplementary Table S2) and the relative distribution of C18 FA (in % of total C18 FA) present at the tube from 0 h and 24 h incubation time as detailed by [7].

The biohydrogenation of dietary unsaturated C18 FA were then calculated as:

(1) $$BH\;of\;FA\;\left(\%\right)=\frac{\left[\mathrm{FA}0h]\;-\;[\mathrm{FA}24h\right]}{\;\left[\mathrm{FA}0h\right]}\;\times \;100$$

where FA can be *c*9–18:1, *c*9,*c*12–18:2 or *c*9,*c*12,*c*15–18:2 and BH stands for biohydrogenation.

All C18 FAs that displayed a positive balance during the 24 h of incubation were considered here as biohydrogenation products (BHPs). The relative yield of the main classes of BHPs (18:0, 18:1 isomers; 18:2 isomers and oxo-FA) were computed from the C18 FA balance data and expressed in percentage of total BHP, as detailed by [7].

The ratios between BH of each dietary C18 unsaturated FA (*c*9–18:1, *c*9,*c*12–18:2 and *c*9,*c*12,*c*15–18:3) and the total VFA were also computed.

### 2.6. Statistical Analysis

Data were analyzed as a randomized complete block design using the Proc Mixed of SAS (SAS Institute inc, Cary, NC, USA), considering the tube as the experimental unit, and the “incubation run” as a random block according to the following model:

*Yij* = *µ* + *Ti* + *Dj* + *eij* (2)

where *Yij* is the observation, *µ* is the overall mean, *Ti* is the fixed effect of treatment (CT doses), *Dj* is the random effect of incubation run and *eij* is the residual error. Orthogonal contrasts were used to detect linear and quadratic responses. When significant effects of treatments were detected, the least square means were compared using the pairwise Tukey comparison test. Differences were declared significant at *p* < 0.05.

## 3. Results

The net production of the total VFA and of the individual VFA are presented in Table 1. Increasing the dose CT caused a linear decrease in the total VFA production (*p* < 0.001) and in all the individual VFA production, except for iso-5:0, that decreased quadratically (*p* = 0.004). However, the 2:0/3:0 ratio was not influenced by CTs, averaging 3.0. *Cistus ladanifer* CT extract doses did not affect the pH of fluid ruminal at 24h of incubation (*p* < 0.05; average of 6.74).

The BH for dietary unsaturated C18 FAs are presented in Table 2. The BH of *c*9–18:1 and *c*9,*c*12–18:2 did not differ (*p* > 0.05) among treatments. The BH of *c*9,*c*12,*c*15–18:3 decreased linearly with the increasing doses of CT (*p* = 0.003). The CT had no effect on the yield of 18:0 and sum of 18:1 BHP. The yield of 18:0 ranged from 45.6 to 50.2% of total BHP, and the yield of 18:1 BHP ranged 34.0 to 35.9% of total BHP. However, the yield of 18:2 BHP decreased linearly with the increasing dose of CT. The yield of oxo-FA showed a quadratic increase (*p* = 0.001) being higher in treatments with CT (average 14.3%) than in the control (11.5%).

The BH/VFA ratios, showed in Table 2, were calculated in order to evaluate the relation conversely between BH and fermentative activity. The BH/VFA ratios for *c*9–18:1 and *c*9,*c*12–18:2 increased linearly with CT dose, whereas the BH/VFA ratio for *c*9,*c*12,*c*15–18:3 was unaffected by CT dose.

Table 3 shows the effect of increasing doses of CT on the balance of C18 FA during the 24 h of incubation. The *c*9–18:1; *c*9,*c*12–18:2 and *c*9, *c*12,*c*15–18:3, the main dietary unsaturated C18 FAs, displayed large negative balances (losses) in all treatments. The losses of *c*9–18:1; *c*9,*c*12–18:2 and *c*9,*c*12,*c*15–18:3 did not differ among treatments (*p* > 0.05).

The C18 FAs displaying a positive balance (i.e., biohydrogenation products-BHP) comprise the 18:0, 18:1 and 18:2 isomers and oxo-FA. Occasionally, other C18 FAs (i.e., *c*11–18:1) presented negative balances, but the overall pattern presented positive balances. Stearic acid (18:0) varied from 656 µg/tube to 774 µg/tube and did not differ significantly among the CT doses tested. All 18:1 isomers, except *c*11–18:1, displayed consistently positive balances that were larger for *t*11–18:1, from 324 µg/tube to 456 µg/tube, and was not affected by CT. The *t*4-, *t*5-, *t*6- + *t*7- + *t*8-, *t*15- and *t*16 + *t*14–18:1 isomers increased linearly with the increasing CT doses, whereas the *t*9–18:1 isomer decreased linearly, and none of the other 18:1 isomers identified was significantly affected by *C. ladanifer* CT extract (*p* > 0.05).

Collectively, the 18:2 isomers changed from 37 µg/tube (in 75 and 100 g/kg DM of ***C.*** *ladanifer* CT extract) to 69 µg/tube of the total BHP formed (in the control). The 18:2 isomers showed a quadratic response, with highest values in the control (*p* = 0.018) than in CT treatments. The major individual 18:2 isomer was the *t*9, *c*12–18:2, which ranged from 9.4 µg/tube to 21.5 µg/tube and presented a quadratic effect (*p* = 0.047) similar to the 18:2 isomers sum. The *c*9,*t*11-, *t*9, *t*12- and *c*9, *t*12–18:2 decreased linearly with increasing CT doses. Relative to oxo-FAs, only the 13-oxo-18:0 increased linearly (*p* = 0.019) with CT dose whereas the others oxo-FAs were unaffected.

Table 4 shows the effect of increasing CT doses on the balance of other non-C18 FAs, mostly derived from microbial de novo synthesis, and DMAs during the 24 h of incubation. Most of these FAs presented a positive balance, except for 12:0, 20:1 and *c*7–16:1. The 14:0, 16:0, cyclo-17:0 and 26:0 FA decreased linearly with the CT dose, whereas the 28:0 and *c*7–16:1 presented a quadratic effect, with lower values for 50 and 75 g/kg DM of *C. ladanifer* CT extract in 28:0, and for 50 g/kg DM of *C. ladanifer* CT extract in *c*7–16:1.

All the odd- and branched-chain FAs (OBCFAs) were affected by CT and showed a similar trend, decreasing linearly with the CT dose, with exception for the 13:0, which showed a quadratic response, with higher value in the control (without CT supplementation) and lower in 75 g/kg DM of *C. ladanifer* CT extract.

The total of DMAs comprised from 255 to 721 µg/g of DM and presented a quadratic response to the CT dose. All of the DMAs decreased linearly with the CT dose, except for 18:1, which presented a quadratic response, with a lower value in the 75 g/kg DM of *C. ladanifer* CT extract.

## 4. Discussion

The utilization of CT extracts from various origins to modulate the ruminal BH as a way to increase the rumen outflow of healthy FA, such as *t*11–18:1, *c*9, *t*11–18:2 and dietary PUFA has been the subject of several studies [19,27,28,29,30]. Although some results have shown the potential of this strategy to improve the FA profile of ruminant edible fat, overall, the effects of CT extracts on ruminal metabolism including fermentation [31,32,33,34,35] and BH [5,6,7,12,13,19,27,29,30] are inconsistent. The diversity the effects of CT extracts on ruminal fermentation and BH may be due to several factors, such as CT composition and concentration, basal substrate, and experimental conditions [19,33,34]. In the current study, the increasing doses of *C. ladanifer* CT reduced the VFA production, which is in disagreement with previous in vitro studies where 100 g/kg DM of *C. ladanifer* CT extract did not depress ruminal fermentation [7,12]. The effects on ruminal BH were also inconsistent with previous in vitro studies using *C. ladanifer* CT extracts, where the incubation of 100 g/kg DM of extract increased the *c*9,*c*12–18:2 and *c*9,*c*12,*c*15–18:3 BH and the *t*11-18:1 production [7,12]. Conversely, in the present work, the incubation of a *C. ladanifer* CT extract at doses between 25 to 100 g/kg DM had a limited effect on BH, affecting only *c*9,*c*12,*c*15-18:3 BH that decreased and the production of the minor BHP, without a change to *t*11-18:1 production. *Cistus ladanifer* CT extract used in the present work was obtained from the same *C. ladanifer* plants and by the same extraction methodology used in Guerreiro et al., [7]. The tested doses were the same or below to the dose used previously [7,12]. However, in the present experiment, different in vitro incubation conditions, such as basal substrate and incubation time, were used, which may help to explain the difference in results on fermentation and BH.

The composition of the basal diet is appointed as one of the reasons for the inconsistent effect of plant secondary metabolites, such as tannins on ruminal fermentation and BH [19,34,35]. In the present work, the substrate consisted of a high-concentrate diet while in previous works, with C. *ladanifer* CT extract, 70% of the substrate was composed of dehydrated Lucerne [7,12]. A shift in the microbial composition and activity associated to substrate composition might have led to distinct fermentation and BH responses to *C. ladanifer* CT extract [36,37]. Moreover, multiple interactions that might occur between *C. ladanifer* CT and other dietary components [19,30] can contribute to the discrepancy among in vitro experiments with *C. ladanifer* CT extracts. In vivo trials with lambs showed that the effect of the *C. ladanifer* plant (leaves and soft stems) in ruminal BH is dependent on the characteristics of the basal diets, such as concentrate level and composition, and lipid supplementation [28]. An increase in the *t*11–18:1 and a reduction in 18:0 contents in abomasal digesta was observed when *C. ladanifer* (250 g/kg DM) was included in a high-forage diet supplemented with vegetable oils rich in PUFA (60 g/kg DM), but not when it was incorporated in a lipid unsupplemented diet [15]. On the other hand, the incorporation of *C. ladanifer* (50 and 200 g/kg DM) in a diet containing 1:1 forage-to-concentrate supplemented with 0, 40 or 80 g/kg of vegetable oils increased the ruminal production of *t*10-18:1 instead *t*11–18:1, which was exacerbated by the simultaneous inclusion of PUFA and *C. ladanifer* in diets [18]. Moreover, in a diet containing 1:1 forage-to-concentrate supplemented with 60 g/kg of vegetable oil in which the cereal was replaced by dehydrated citrus pulp, the *C. ladanifer* (150 g/kg) had a minor effect on the fatty acid composition of intramuscular fat [38]. Conversely, when combined with cereals, *C. ladanifer* led to an increase in the 18:0 and reduced the BH intermediates in intramuscular fat, suggesting that under these conditions. *C. ladanifer* increases the ruminal BH completeness [38]. As suggested by the in vivo experiments with the *C. ladanifer* plant, interactions between the basal substrate and *C. ladanifer* CT may have occurred, resulting in distinct CT responses when the composition of the basal diet is changed.

Incubation time should also be considered in the interpretation of the present results. Relative to previous in vitro trials with *C. ladanifer* CT extracts, in the present work, a longer incubation time was used (6 h vs. 24 h). In long incubation times, similar BH endpoints might be achieved independently to incur differences among treatments at earlier stages of the incubation [39]. Unfortunately, the FA profile in ruminal fluid was not monitored during incubation, and the possibility of the *C. ladanifer* CT response varying over incubation time cannot be excluded.

Odd- and branched-chain FAs (OBCFAs) in rumen are mainly present in membrane lipids of rumen microbes, which are able to synthetize them from propionate and/or branched-chain VFAs derived from branched-chain amino acids, and incorporate them into their cell membranes [40]. Rumen bacterial lipids are also characterized by a high content of plasmalogens, a subclass of phospholipids containing alk-1-enyl (vinyl) ether chains which can be detected by the presence of DMA released under acid catalysis [26]. Thus, OBCFAs and DMAs have been proposed as microbial markers in the rumen ecosystem [41,42,43]. The general decrease in OBCFA and DMA production with increasing doses of *C. ladanifer* CT associated with a reduction in VFA production suggests a reduction of bacterial activity. In fact, CT has a inhibitory effect on the activity and growth of microorganisms, which is probably due to their ability to form complexes with various types of molecules such as proteins, polysaccharides and minerals, allowing them to interact with microorganisms’ membranes, cell walls and extracellular structures and also reduce nutrient availability [44].

The inhibitory effects of *C. ladanifer* CT were more pronounced for OBCFA and DMA production (up to a 65% reduction with 100 g/kg DM of *C. ladanifer* CT), than for the total VFA production (a 27% reduction with 100 g/kg DM of *C. ladanifer* CT). Moreover, the BH of *c*9-18:1 and *c*9,*c*12–18:2 was not affected by increasing doses of *C. ladanifer* CT, although the BH of *c*9,*c*12,*c*15–18:3 was depressed. The basal substrate was supplemented with 60 g/kg of sunflower oil, which contains *c*9,*c*12–18:2 as the main FA, followed by *c*9–18:1, while *c*9,*c*12,*c*15–18:3 is absent from sunflower oil and thus it was provided in small amounts from feed structural lipids. Thus, the availability of *c*9,*c*12,*c*15–18:3 and its subsequent BH was dependent on feed material degradation, and closely followed the VFA production, as it is evidenced by constant ratio between *c*9,*c*12,*c*15–18:3 BH and VFA production.

Thus, it is apparent that there was an uncoupling between *c*9–18:1 and *c*9,*c*12–18:2 BH activity and other microbial metabolic activities, but within the other microbial metabolic activities, and also between the ATP energy yielding catabolism (i.e., VFA production) and microbial growth (i.e., OBCFA and DMA production). The ATP yield is expected to be closely related to the VFA produced, as VFAs are end-products of the fermentative pathways [45]. The ATP obtained is expected to support the microbial growth. The uncoupling between energy yield and growth means that energy is being produced (i.e., VFAs) but not translated in obvious growth (i.e., evaluated by the yield in microbial markers such as BCFAs and DMAs). Polyunsaturated fatty acids (PUFAs) have well established bacteriostatic effects and it is reported that, in pure cultures studies, *Butyrivibrio fibrisolvens* growth was initiated only when PUFAs (*c*9,*c*1218:2 and *c*9,*c*12,*c*15–18:3) had been metabolized and reduced to *t*11–18:1 [46]. The presence of a high concentration of CT can also exert disruptive effects on microbial cell membranes [44,47]. Some bacteria are able to handle toxic environmental stimuli by increasing the *trans*-18:1 esterified in cell membrane phospholipids and thus diminishing its fluidity [48]. Moreover, *trans*-18:1 has also been reported as a growth promoter for some bacteria [49]. Thus, rumen BH might confer protection to rumen microbiota subjected to high PUFA and high CT concentrations by decreasing the PUFA concentrations and simultaneously by increasing the availability of *trans*-18:1 to be incorporated in rumen microbiota cell membranes [9]. In the present experiment, the substrate incubated contained a high PUFA concentration (i.e., 60 g/kg of sunflower oil) and increasing amounts of CT. So, in response to these adverse conditions, the microbial population may have to limit growth and fermentative activity while maintaining the BH pathways in order to reduce the concentration of PUFAs and generate *trans*-18:1 and incorporate it into their membranes. The maintenance of a higher BH activity, compared to growth and fermentative activity, in response to increasing doses of CT is consistent with the hypothesis that BH is an adaptive stress response against not only a high PUFA concentration but also against other membrane disruptors, such as CT. The disruptive effects of CTs on the cell membrane might also explain the apparent uncoupling between ATP production and growth observed in the present experiment, as increased energy would be used for the maintenance of ion gradients across the damaged cell membrane [50].

Condensed tannins have been reported to inhibit the last step of BH, decreasing the 18:0 formation and leading to *t*11-18:1 accumulation [5,6,11]. In the present study, no effect of *C. ladanifer* CT on 18:0 and on 18:1 isomers yield was observed, which confirms previous observations with the same CT source [7,12,13]. In fact, increasing doses of *C. ladanifer* CT did not modify the major BHP, despite the small but significant increase in oxo-FA and decrease in 18:2 BHP. The 10-oxo-18:0 and 13-oxo-18:0 have been described as end products of the BH of the *c*9–18:1 and *c*9,*c*12–18:2, respectively [26,51,52]. In the present experiment, oxo-FA represented up to 15.4% of the BHP, which is consistent with previous reports [7,12,13].

## 5. Conclusions

The incubation of increasing doses of *C. ladanifer* CT extract (0, 25, 50, 75 and 100 mg/g kg DM) using oil-supplemented high-concentrate subtract led to a moderate decrease in VFA production and to a very pronounced depression of OBCFA and DMA production, without affecting the BH or the BH products yield. The apparent uncoupling between growth, ATP production and BH activity could be the reflex of the response of the microbial population to stress induced by high CT and PUFA concentration in the rumen. In addition to assessing the impact of increasing doses of CT extract from *C. ladanifer* on ruminal fermentation and BH, it was also our objective determine the *C. ladanifer* CT extract doses which optimize the *c*9,*t*11–18:2 and *t*11–18:1 production. However, in this in vitro experiment, it was not possible achieve this goal. The present results emphasize the inconsistency regarding the effects of CT on rumen fermentation and BH. Further studies are needed to elucidate the impact of CT on the ruminal population and metabolism for the adequate use of CT extract as a feed additive to improve the nutritional value of ruminant fat.

## Figures and Tables

**Table 1:** Effect of increasing doses of *Cistus ladanifer* condensed tannin (CT) extract (0, 25, 50, 75 and 100 g/kg dry matter (DM)) on net production of volatile fatty acid (VFA, mmol/L) during the 24 h of incubation.

	*C. ladanifer* CT, g/kg Dry Matter					SEM	*p* Values	
	0	25	50	75	100		Linear	Quadratic
Total VFA	35.4	33.2	32.6	26.6	25.8	1.59	<0.001	0.629
2:0	22.0	20.9	20.7	16.4	16.3	1.20	<0.001	0.623
3:0	7.28	6.79	6.62	5.60	5.19	0.314	<0.001	0.554
iso-4:0	0.41	0.24	0.26	0.20	0.16	0.042	<0.001	0.219
4:0	4.43	4.33	4.15	3.68	3.45	0.205	<0.001	0.190
iso-5:0	0.61	0.46	0.41	0.36	0.33	0.025	<0.001	0.004
5:0	0.50	0.40	0.37	0.30	0.27	0.020	<0.001	0.107
2:0/3:0 ratio	3.04	3.09	3.02	2.92	3.13	0.138	0.734	0.738

SEM, standard error of the mean.

**Table 2:** Effect of increasing doses of *Cistus ladanifer* condensed tannin (CT) extract (0, 25, 50, 75 and 100 g/kg DM) on dietary C18 unsaturated fatty acids biohydrogenation (BH), relative yield in the BH products, and on BH/volatile fatty acid (VFA) ratio.

	*C. ladanifer* CT, g/kg Dry Matter					SEM	*p* Values	
	0	25	50	75	100		Linear	Quadratic
Biohydrogenation (%)								
*c*9–18:1	51.2	52.4	47.1	48.6	48.5	4.30	0.426	0.760
*c*9,*c*12–18:2	68.7	67.1	63.4	64.6	65.0	3.93	0.431	0.545
*c*9,*c*12,*c*15–18:3	67.6	61.2	52.3	54.7	50.8	3.76	0.003	0.239
Biohydrogenation Products (%)								
18:0	49.2	47.8	45.6	50.2	49.5	3.50	0.666	0.327
18:1 ^1^	35.8	35.3	35.9	34.0	35.0	4.65	0.553	0.952
18:2 ^2^	4.18	3.36	3.44	2.36	2.34	0.608	0.020	0.841
oxo-FA ^3^	11.5	14.1	15.4	13.9	13.6	2.15	0.046	0.001
Biohydrogenation:VFA ratio								
*c*9–18:1BH/VFA	1.47	1.59	1.45	1.86	1.95	0.182	0.009	0.340
*c*9,*c*12–18:2BH/VFA	1.95	2.03	1.95	2.47	2.58	0.171	0.004	0.278
*c*9,*c*12,*c*15–18:3BH/VFA	1.93	1.85	1.60	2.10	2.01	0.159	0.437	0.235

SEM, standard error of the mean.^1^ Sum of 18:1 isomers, all 18:1 biohydrogenation intermediates, i.e., all 18:1 FA listed in the Table 3; ^2^ Sum of 18:2 isomers, all 18:2 biohydrogenation intermediates, i.e., all 18:2 FA listed in the Table 3; ^3^ Sum of 10-oxo-18:0 and 13-oxo-18:0.

**Table 3:** Effect of increasing doses of *Cistus ladanifer* condensed tannins (CT) extract (0, 25, 50, 75 and 100 g/kg DM) on net C18 fatty acid changes (µg/tube) during the 24 h of incubation.

	*C. ladanifer* CT, g/kg Dry Matter					SEM	*p* Values	
	0	25	50	75	100		Linear	Quadratic
C18 FA loss								
*c*9-18:1	−635	−646	−576	−597	−597	57.5	0.479	0.730
*c*9,*c*12-18:2	−1004	−946	−827	−896	−956	240.2	0.617	0.230
*c*9,*c*12,*c*15-18:3	−23.5	−21.5	−16.9	−19.4	−18.4	3.61	0.061	0.233
Sum	−1663	−1613	−1420	−1512	−1572	259.4	0.544	0.364
C18 FA gains								
18:0	774	750	656	743	753	100.1	0.836	0.380
18:1 isomers								
*t*4-	2.84	3.33	3.60	4.44	4.68	0.718	<0.001	0.947
*t*5-	1.25	0.93	2.08	2.40	3.27	0.815	0.002	0.395
*t*6-, *t*7-, *t*8-	22.8	27.1	27.1	31.5	33.3	4.73	0.009	0.960
*t*9-	61.7	54.7	43.4	42.2	40.2	6.40	<0.001	0.538
*t*10-	33.6	40.7	40.4	42.5	41.8	7.18	0.276	0.493
*t*11-	456	397	324	333	376	136.9	0.186	0.155
*t*12-	27.4	29.8	28.6	32.2	33.7	4.79	0.131	0.780
*t*15-	14.3	16.4	15.1	19.0	20.7	3.08	0.025	0.566
*c*11-	−10.6	−11.5	−8.12	−10.0	−9.42	2.90	0.518	0.747
*c*12-	22.2	21.2	18.5	20.0	21.2	4.23	0.695	0.391
*c*13-	1.16	1.05	0.35	1.75	1.34	0.289	0.268	0.180
*t*16-, *t*14-	12.5	15.8	14.3	16.9	18.3	2.75	0.028	0.951
*c*16-	2.99	3.29	3.09	2.92	3.39	0.678	0.634	0.736
Sum	654	607	522	544	593	153.2	0.371	0.227
18:2 isomers								
*t*9,*t*12-	9.47	6.33	5.34	4.18	3.47	0.677	<0.001	0.080
*c*9,*t*12-	17.1	13.4	11.0	8.69	6.86	2.33	<0.001	0.543
*t*11,*c*15-	1.27	1.33	1.07	1.11	0.62	0.343	0.101	0.437
*c*9,*t*11-	8.02	3.83	0.26	0.57	1.39	2.178	0.022	0.092
CLA *tt*	12.3	12.7	10.8	11.9	14.9	3.18	0.536	0.336
*t*9,*c*12-	21.5	15.4	12.9	10.3	9.36	2.80	<0.001	0.047
Sum	69.0	53.0	41.4	36.8	36.5	8.83	<0.001	0.018
Oxo FA								
10-oxo-18:0	147	180	175	163	162	17.9	0.819	0.280
13-oxo-18:0	24.1	30.6	30.0	30.8	31.8	5.09	0.019	0.192
Sum	171	210	205	194	194	19.9	0.354	0.273
Total gain	1663	1613	1420	1512	1572	259.4	0.544	0.364

SEM, standard error of the mean.

**Table 4:** Effect of increasing doses of *Cistus ladanifer* condensed tannin (CT) extract (0, 25, 50, 75 and 100 g/kg DM) on other fatty acids (FAs) and dimethyl acetals (DMAs) balance (ug/g DM). OBCFAs: odd- and branched-chain FAs.

	*C. ladanifer* CT, g/kg Dry Matter					SEM	*p* Values	
	0	25	50	75	100		Linear	Quadratic
FA								
12:0	−99.8	−96.7	−146	−79.7	−103	34.67	0.921	0.619
14:0	190	181	85.3	98.6	62.0	26.6	<0.001	0.591
16:0	1456	1274	316	785	329	347.1	0.021	0.507
Cyclo-17:0	37.0	54.9	23.2	10.4	−10.2	13.10	0.003	0.252
20:0	50.6	47.3	13.3	37.4	22.4	13.69	0.141	0.207
20:1	−7.38	−6.85	−18.8	−10.7	−11.3	4.524	0.423	0.307
21:0	0.94	3.03	0.34	−0.80	3.64	1.32	0.707	0.213
22:0	43.9	37.1	-3.93	21.0	14.0	15.99	0.149	0.056
23:0	9.74	5.80	2.30	6.50	4.99	2.708	0.316	0.229
24:0	39.1	34.3	10.4	25.5	10.4	10.03	0.050	0.629
26:0	164	112	72.0	87.5	69.1	17.32	<0.001	0.073
28:0	8.38	10.8	0.44	0.06	10.5	3.151	0.521	0.039
*c*7-16:1	−1.77	−1.45	−11.82	−5.74	−5.67	4.276	0.063	0.041
*c*9-17:1	1.51	−0.80	−2.24	1.69	−2.36	2.006	0.417	0.805
OBCFAs								
13:0	15.8	7.50	9.09	4.30	7.48	2.093	0.005	0.036
i-14:0	28.9	26.6	19.9	16.1	14.0	5.33	0.027	0.879
i-15:0	64.4	45.5	24.7	21.6	9.42	5.06	<0.001	0.062
a-15:0	139	124	75.1	71.2	50.7	9.13	<0.001	0.334
15:0	130	111	58.0	61.6	43.8	13.5	<0.001	0.251
i-16:0	44.2	47.8	32.2	26.7	20.2	5.36	<0.001	0.558
i-17:0	46.8	35.8	22.7	30.6	18.6	5.36	0.001	0.322
a-17:0	72.5	58.1	38.0	42.3	20.8	9.61	<0.001	0.739
17:0	73.4	52.3	31.4	33.8	23.5	10.14	0.001	0.228
Total	615	508	311	308	209	52.4	<0.001	0.301
DMAs								
12:0	16.2	17.2	11.1	6.46	2.93	2.774	<0.001	0.327
i-14:0	18.5	13.3	0.63	3.00	−2.44	3.587	<0.001	0.254
14:0	31.0	23.3	11.3	5.47	−6.31	5.258	<0.001	0.911
i-15:0	10.2	13.1	5.94	3.58	−0.41	2.528	<0.001	0.300
a-15:0	528	392	260	183	221	54.79	<0.001	0.051
15:0	9.53	8.36	2.64	1.83	0.51	2.235	0.002	0.588
16:0	68.5	55.9	40.7	37.0	18.4	6.28	<0.001	0.983
18:0	9.80	4.18	4.59	3.28	3.80	1.864	0.018	0.087
18:1	29.3	19.3	6.46	18.4	16.8	3.45	0.020	0.003
Total	721	546	343	262	255	58.6	<0.001	0.041

SEM, standard error of the mean.

## Data Availability

Data sharing not applicable.

## Supplementary Materials

The following are available online at https://www.mdpi.com/2076-2615/11/3/761/s1, Table S1: Initial (0 h) and final (24 h) concentration of total volatile fatty acids (VFA, mmol/L), molar percentages of individual VFA (mol/100 mol) and pH during the in vitro rumen incubation with 0, 25, 50, 75 and 100 g/kg DM of *Cistus ladanifer* condensed tannins extract, Table S2: Initial (0 h) and final (24 h) concentration of C18 fatty acids (FA, µg per tube) during the in vitro rumen incubation with 0, 25, 50, 75 and 100 g/kg DM of *Cistus ladanifer* condensed tannins extract.
