# Supplementary material for: Effects of Increasing Doses of Condensed Tannins Extract from Cistus ladanifer L. on In Vitro Ruminal Fermentation and Biohydrogenation
Source: Animals (Basel). 2021 Mar 10;11(3):761. doi: 10.3390/ani11030761 (PMC7998652; doi:10.3390/ani11030761)
Supplement: Supplementary file 1 [file animals-11-00761-s001.pdf]

Table S1: Initial (0 h) and final (24 h) concentration of total volatile fatty acids (VFA, mmol/L), molar percentages of individual VFA (mol/100 mol) and pH during the *in vitro* rumen incubation with 0, 25, 50, 75 and 100 g/kg DM of *Cistus ladanifer* condensed tannins extract

	0	25	50	75	100	SEM	*P* values	
							Linear	Quad.
Initial (0 h)								
Total VFA	16.8	18.4	19.3	17.8	18.7	1.03	-	-
2:0	61.5	62.9	65.1	62.9	62.7	0.69	-	-
3:0	16.2	16.1	15.1	16.2	16.8	0.46	-	-
iso-4:0	2.59	2.39	2.12	2.30	2.48	0.193	-	-
4:0	11.8	11.2	10.8	11.2	10.9	0.434	-	-
iso-5:0	3.20	2.93	2.73	2.92	2.86	0.199	-	-
5:0	2.45	2.31	2.08	2.29	2.22	0.107	-	-
pH, 0 h	6.89	6.94	6.97	6.99	7.01	0.015	-	-
Final (24 h)								
Total VFA	52.2	51.6	51.9	44.4	44.5	1.74	<0.001	0.279
2:0	62.1	63.2	64.1	62.2	63.0	0.529	0.546	0.121
3:0	19.2	18.9	18.4	19.1	18.8	0.433	0.412	0.239
iso-4:0	1.61	1.32	1.30	1.37	1.38	0.075	0.023	0.002
4:0	12.3	12.4	12.1	12.8	12.4	0.438	0.510	0.993
iso-5:0	2.20	1.92	1.80	1.97	1.95	0.086	0.016	0.001
5:0	1.75	1.60	1.48	1.58	1.54	0.049	0.003	0.009
pH, 24 h	6.73	6.75	6.74	6.73	6.77	0.039	0.417	0.672

SEM, Standard error of the mean.

Table S2: Initial (0 h) and final (24 h) concentration of C18 fatty acids (FA, µg per tube) during the *in vitro* rumen incubation with 0, 25, 50, 75 and 100 g/kg DM of *Cistus ladanifer* condensed tannins extract

	0	25	50	75	100	SEM	*P* values	
							Linear	Quad.
C18 FA, 0 h								
18:0	506	513	504	498	520	37.6	-	-
*t*11-18:1	44.3	44.3	41.4	42.2	44.1	4.71	-	-
*c*9-18:1	1245	1243	1177	1239	1233	60.2	-	-
*c*9,*c*12-18:2	1388	1411	1285	1413	1422	323.7	-	-
*c*9,*c*12,*c*15-18:3	33.6	34.8	31.9	35.6	35.0	4.79	-	-
c9,*t*11-18:2	4.91	4.06	5.64	6.34	6.02	2.136	-	-
Total C18 FA	3391	3348	3418	3423	3510	462.5	-	-
C18 FA, 24 h								
18:0	1280	1263	1160	1241	1273	112.1	0.888	0.367
*t*11-18:1	500	441	366	375	420	139.4	0.184	0.149
*c*9-18:1	610	598	602	643	635	54.1	0.421	0.732
*c*9,*c*12-18:2	383	466	458	517	467	108.0	0.218	0.340
*c*9,*c*12,*c*15-18:3	10.1	13.3	15.0	16.2	16.6	1.78	<0.001	0.153
c9,*t*11-18:2	12.9	7.89	5.91	6.91	7.41	3.447	0.062	0.065
Total C18 FA	3209	3311	2819	3207	3166	358.8	0.774	0.454

SEM, Standard error of the mean.
